# Gas-Producing Renal Infection Presenting as Pneumaturia: A Case Report

**DOI:** 10.1155/2013/730549

**Published:** 2013-04-22

**Authors:** Youssef S. Tanagho, Jonathan M. Mobley, Brian M. Benway, Alana C. Desai

**Affiliations:** Division of Urologic Surgery, Department of Surgery, Washington University School of Medicine, 4960 Children's Place, Campus Box 8242, Saint Louis, MO 63110, USA

## Abstract

We present a case of persistent pneumaturia of one-year duration in a fifty-five-year-old male with a history of spinal cord injury. The evaluation demonstrated gas throughout the collecting system attributable to a urinary tract infection with a gas-forming organism, *Klebsiella pneumoniae*.

## 1. Introduction

Pneumaturia, defined as the passage of “gas” in the urine, is the result of gas in the urinary tract and can be due to recent instrumentation, fistulae into the bladder or upper urinary tract from the bowel or vaginal canal (commonly associated with diverticulitis, malignancy, or trauma), urinary diversion, renal tumor infarction, or, as in this case, urinary tract infection with a gas forming organism. Evaluation of pneumaturia may include cystoscopy, colonoscopy, CT of the abdomen/pelvis, and barium or hypaque enema. Herein, we present a case of long-standing pneumaturia in which imaging revealed gas within the collecting system and subsequent evaluation demonstrated that a gas-forming organism was the etiological agent. Treatment was uncomplicated.

## 2. Case Presentation

**Figure 1:**
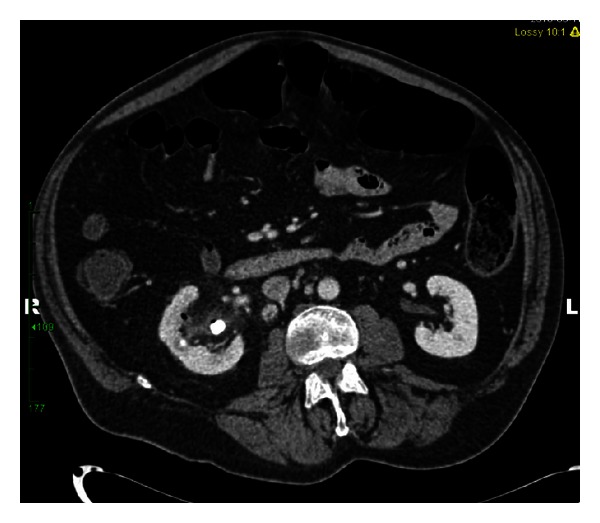
CT abdomen with contrast demonstrates right nephrolithiasis, stranding of the renal pelvis, and gas within the right collecting system.

**Figure 2:**
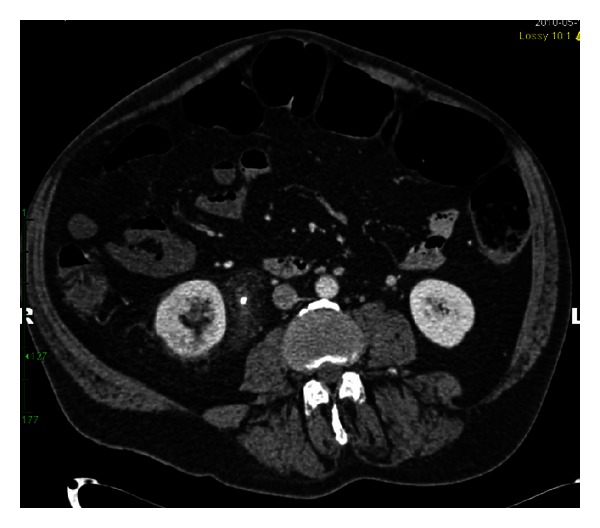
CT abdomen with contrast demonstrates a wedge-shaped segment of low attenuation in the posterior, lower pole of the right kidney consistent with pyelonephritis. Please note the retained right ureteral stent and periureteral stranding.

**Figure 3:**
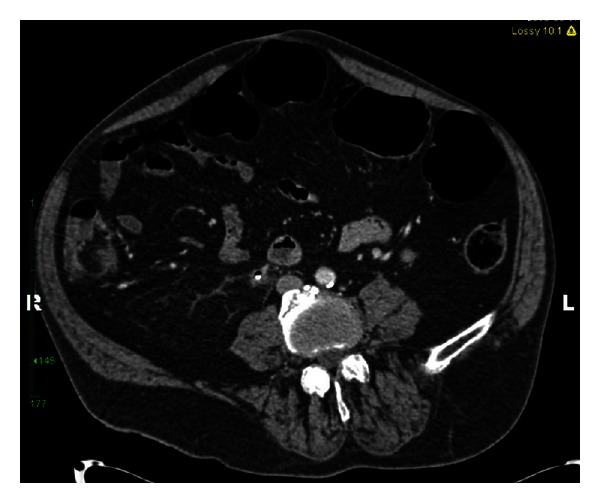
The CT image demonstrates a retained right ureteral stent and the presence of gas in the right ureter.

**Figure 4:**
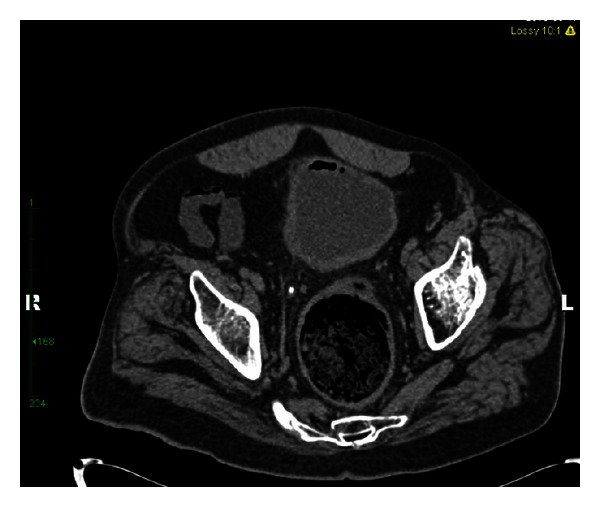
CT pelvis illustrates the presence of gas in the lumen of the bladder. There is no presence of gas in the wall of the bladder to suggest emphysematous cystitis.

RM is a 55-year-old, nondiabetic, male with a history of neurogenic bladder secondary to spinal cord injury (SCI). He was diagnosed with a 3 cm staghorn calculus approximately 18 months prior to presentation. He underwent left ureteral stent placement followed by shock wave lithotripsy by a community urologist. He was lost to follow-up and presented to our institution with recurrent urinary tract infections and a retained ureteral stent. At the time of his office visit, he reported intermittent hematuria, occasional, mild abdominal pain, and persistent pneumaturia for the past one year. He denied any fever or fecaluria over the past year. There was no history of bowel disease or pelvic irradiation. A CT urogram was obtained (Figures 1, 2, 3, and 4), which showed gas within the collecting system and a wedge-shaped segment of low attenuation in the lower pole of the right kidney, consistent with pyelonephritis. Urine culture obtained in the office grew *Klebsiella pneumoniae*. The patient was started on a 2-week course of Ciprofloxacin, and a percutaneous nephroureteral stent was placed in anticipation of definitive stone management with percutaneous nephrolithotomy. A urine culture was obtained from the renal pelvis at the time of nephrostomy tube placement. The renal pelvis urine culture was negative. However due to a high risk of infection-related complication, the patient was continued on Ciprofloxacin empirically until the time of surgery. RM underwent right percutaneous nephrolithotomy without complications, although difficult stent removal due to encrustation resulted in prolongation of the case by an additional 1.5 hours. Renal stone fragments were sent for culture and revealed no growth. Stone composition was a mixture of calcium phosphate (75%) and calcium oxalate monohydrate (15%). CT obtained on postoperative day one showed a residual 9 mm fragment in the upper pole and a 2 mm fragment in the renal pelvis. The patient underwent second look nephroscopy three days following the initial surgery to retrieve the residual fragments. He developed fever to 38.6°C (101.5°F) on post-operative day number one without hypotension or tachycardia. Urine and blood cultures obtained at that time were negative. The patient's post-operative course was otherwise uneventful, and he was discharged on postoperative day three following second look nephroscopy with a nephrostomy tube to gravity drainage. The nephrostomy tube was kept in place for two weeks, at which time an antegrade nephrostogram was performed and the stent was internalized. The internal ureteral stent was removed one week later. Of note, the patient reported that his pneumaturia resolved approximately one week after starting the course of antibiotics.

## 3. Discussion

Pneumaturia, a sign of gas in the urinary tract, can be due to a number of causes, such as enterovesical or vesicovaginal fistulae, iatrogenic causes, emphysematous cystitis, and, less commonly, emphysematous pyelonephritis. Emphysematous cystitis is an infection of the bladder wall, while emphysematous pyelonephritis is an infection of the renal parenchyma. Management of emphysematous cystitis includes early diagnosis, administration of broad-spectrum antibiotics, strict diabetic control, and urinary drainage [1]. Delay in diagnosis may contribute to the 20% mortality rate associated with this condition [2]. Emphysematous pyelonephritis is a necrotizing infection of the renal parenchyma, the vast majority of cases occurring in patients with poorly controlled diabetes mellitus [3].

In a retrospective review of 38 patients, Wan et al. elucidated two types of emphysematous pyelonephritis. Type I is characterized by parenchymal destruction with either absence of fluid collection or the presence of streaky or mottled gas; this pattern was found to be associated with a more fulminant clinical course, with a 69% mortality rate. Type II may present with renal or perirenal fluid collections or gas within the collecting system and is associated with a significantly decreased mortality risk compared to Type I [4, 5]. All patients in this study in whom there were concomitant stones (27%) had Type II emphysematous pyelonephritis [4]. The radiographic finding of the presence of fluid may represent adequate inflammatory response and vascular supply [4]. This concept was first noted during liver imaging, wherein an alveolar gas pattern without fluid content was found to be a poor prognostic sign for patients with gas-containing liver abscesses [6]. 

The most common findings associated with emphysematous pyelonephritis include fever, flank pain, and pyuria [7]. Lactate-fermenting organisms capable of producing gas include *E. coli*, *Klebsiella pneumonia*, *Proteus*, *Candida,* and *Clostridium* [8]. Emphysematous cystitis or emphysematous pyelonephritis should be suspected in patients with pneumaturia, especially if diabetic. Pneumaturia in the setting of emphysematous pyelonephritis occurs if gas extends into the collecting system. The mainstay of treatment begins with prompt recognition, followed by administration of broad-spectrum antibiotics and urinary drainage with a Foley catheter and percutaneous nephrostomy tube(s) if gas is confined to the collecting system or the patient has an adequate response to antibiotic therapy [7]. If the patient does not respond to conservative management or is clinically worsening, urgent nephrectomy may be required [9].

Historically, urgent nephrectomy was the treatment of choice for emphysematous pyelonephritis. However, recent evidence suggests that percutaneous nephrostomy drainage may be useful in the management of this condition in patients too ill to undergo surgical intervention and as an adjunct if definitive nephrectomy is required [10]. In the case herein, the patient presented with a chronic urinary tract infection, pneumaturia, and Type II emphysematous pyelonephritis. This patient's symptoms of pneumaturia likely represented an improved prognosis consistent with gas in the collecting system as seen in some cases of Type II emphysematous pyelonephritis. Given the improved prognosis of Type II emphysematous pyelonephritis when compared to Type I emphysematous pyelonephritis, it is not surprising to observe that this patient was managed with broad-spectrum antibiotics, decompression of the infected foci with a nephroureteral stent, and definitive stone management [5]. This case illustrates that Type II emphysematous pyelonephritis sometimes can be managed without the need of urgent nephrectomy.

In conclusion, pneumaturia can result from multiple etiologies including enterovesical or vesicovaginal fistulas, iatrogenic causes, emphysematous cystitis, or emphysematous pyelonephritis. We present a rare case of pneumaturia secondary to gas forming *Klebsiella pneumoniae* associated with a staghorn calculus. Historically, emphysematous pyelonephritis was treated with urgent nephrectomy. Recent publications suggest that emphysematous pyelonephritis can be managed with percutaneous drainage and antibiotic coverage in select cases. The patient was successfully managed with percutaneous nephroureteral stent placement followed by subsequent definitive stone management.
